# Foodborne illness among factory workers, Gweru, Zimbabwe, 2012: a retrospective cohort study

**DOI:** 10.1186/s13104-015-1512-2

**Published:** 2015-09-29

**Authors:** Meggie Gabida, Notion T. Gombe, Milton Chemhuru, Lucia Takundwa, Donewell Bangure, Mufuta Tshimanga

**Affiliations:** Department of Community Medicine, University of Zimbabwe, Harare, Zimbabwe; Provincial Medical Directorate, Midlands Province, Ministry of Health and Child Care, Gweru, Zimbabwe

**Keywords:** Food borne illness, Retrospective cohort, *Klebsiella* spp.

## Abstract

**Background:**

On the 20th September 2012 the Gweru district medical officer (DMO) reported a sudden increase in the number of factory workers complaining of symptoms suggestive of gastrointestinal illness. We conducted a retrospective cohort study to determine factors associated with illness among factory workers.

**Methods:**

A retrospective cohort study was conducted from September to October 2012 among 98 randomly selected factory workers. Interviewer administered questionnaires were used to evaluate possible risk factors from which food attack rates, relative risks (RR) and adjusted odds ratios (AOR) were calculated using Epi info version 3.5.1. Bacteriological examination of food samples was performed. In addition rectal swabs and specimens from food handlers and patients were collected for analysis.

**Results:**

Of the 98 workers interviewed, 87/98 (89 %) were males. Consumption of beef stew (AOR = 9.28, 95 % CI 2.78–30.91) was independently associated with foodborne illness. *Klebsiella* spp. were isolated from beef stew and stool specimen of patients. Watery diarrhoea 51/98 (52 %), fatigue 48/98 (49 %) and abdominal cramps 41/98 (42 %) were the most presenting symptoms.

**Conclusions:**

*Klebsiella* spp. was the aetiological agent for the food borne illness at the factory and this resulted from consumption of contaminated beef stew by the workers. As a result of this evidence, the implicated beef was withdrawn from the canteen and the menu cycle was revised to minimise exposure to the same food. Food handlers training in food safety and hygiene and regular canteen inspections for quality assurance were recommended and adopted. No further food borne illness has been reported from the factory.

## Background

Food borne illness is caused by eating or drinking contaminated food or beverage [1] and are caused by pathogens and toxins (chemicals) such as bacteria, viruses and parasites. Foodborne illnesses are the leading cause of illness and death in developing countries [2].

Food can be contaminated across the production chain. Changing patterns in global food production have resulted in food being distributed over large distances and thus contamination is more likely at various points from production to the consumer [3]. Meat products especially, may be contaminated with microorganisms from meat handlers through manufacturing, packaging and marketing. On the other hand, contamination of food products with faecal matter may be the potential source of life threatening food borne illness [3, 4].

Food borne illnesses in humans due to bacterial pathogens and their toxins are well documented worldwide [5]. They impose a substantial societal and economic burden to quality of life by way of acute illnesses. In many parts of the world, public health measures almost eliminated the hazard of contracting food borne illnesses and as a result, for many years the incidence of food borne illnesses was overlooked and it is still underestimated [6].

The global incidence of food borne disease is difficult to estimate, but it has been reported that in 2005 alone, 1.8 million people worldwide died from diarrhoeal diseases. In the United States, foodborne diseases cause an estimated 76 million cases of illness annually [4]. Close to one in five episodes of diarrhoea is likely to be due to food borne disease [3, 4].

Although outbreaks of acute poisoning are frequent in the African region, individual countries have done little to implement surveillance systems for food borne diseases. Data regarding food borne illness in the region is scarce, but studies have shown that the most prevalent pathogens are *Campylobacter*, *Salmonella*, *Shigella*, *Hepatitis*, *Brucella*, *Staphylococcus*, *Bacillus cereus*, *Escherichia coli* and rotavirus [7].

Of the pathogens documented to cause foodborne illnesses, the enterobacteriaceae are the leading cause with staphylococcal intoxication leading in foodborne intoxication. *Salmonella* species has been reported by the United States Department of Agriculture Food Safety and Inspection Service (FSIS) as one of the most common causes of food borne illnesses associated with meat and poultry products [5]. *Salmonella*, *E. coli*, *Proteus* and *Klebsiella* spp. are the most predominant pathogens in food poisoning cases associated with some meat products for instance stewed meant and sausages [8].

In Midlands Province Zimbabwe, diarrheal diseases are captured through the weekly disease surveillance system, and they are among the top five cause of morbidity [9].

The factory where the outbreak occurred is a 24 h production plant with almost 450 workers. The factory provides lunch and super to workers on duty from Monday to Friday. On September 17, 2012, the factory clinic attended to two workers, one male and a female complaining of diarrhoea, nausea, vomiting, fatigue and abdominal cramps. One of the two (female) was a canteen worker who worked in the junior canteen. On September 18, 2012, seven more cases had been attended to by the afternoon complaining of the same symptoms. The illness toll rose from ten cases to 52 cases on September 19, 2012, prompting the factory management to close all water points and provided bottled water to workers. Water points were closed based on the factory management initial hypothesis that water was associated with the illness at the factory. A team from the district medical officer’s (DMO’s) office which included the public health officer visited the factory to assess the situation. On the day of visit, forty-one sick employees were seen during working hours waiting to get treatment at the factory clinic. At the time of assessment no deaths had been recorded.

The purpose of this investigation was to establish the risk factors associated with the foodborne illness at the factory. We hypothesized that consumption of factory water was not associated with foodborne illness.

## Methods

A retrospective cohort study was conducted at a factory where a total of 439 workers are employed. The inclusion criterion for the study was all workers who were on duty and ate or did not eat food from the factory canteen from September 14 to 17, 2012. The exclusion criterion was workers who were not on duty and did not eat from the factory canteen from September 14 to 17, 2012.

The factory employee shift register was used as the sampling frame for participants. We first stratified participants into subgroups according to occupation and calculated proportions using the number of workers per grade as numerator and total number of workers as denominator. The factory uses the Patterson grading system, and in this study, there were four different grades namely the non skilled/general hands (A1–A3), the semi-skilled such as security guards (B1–B3), the skilled including assistant technicians (C1–C4) and the highly skilled including officers and managers (C5–D5). The rationale for adopting the grading system was that menus served at the canteen are based on grades where from A1 to C4 workers eat either beef stew or chicken stew and sadza, alternating everyday (commonly known as traditional diet) and the food is served in the junior canteen. The C5–D5 workers eat a variety of food choices without the stews (commonly known as western diet) and the food is served in the senior canteen. Workers on the shift register were randomly selected for interview using the random number function of a scientific calculator. Unavailable selected participants were replaced by any consenting member in the same grade on duty.

Menus of food served from September 14 to 17, 2012 were verified with the canteen menu cycle. Evaluation of the timeliness, quality of preparedness, outbreak detection, investigation and response was done using a standard checklist adapted from the integrated disease surveillance and response (IDSR) technical guidelines [10].

An environmental assessment was carried out in the factory canteen, where food samples consumed for lunch and supper on September 14–17, 2012 were collected for bacterial culture to identify the source of infection. Specimens including canteen surface swabs, hand and rectal swabs from food handlers and rectal swabs from patients were collected. Water samples from different water points within the factory premises were also collected for microbiological and chemical analysis.

A total of 98 workers were interviewed using a structured questionnaire adapted from the standardized questionnaire developed by the Minnesota Department of Health [11].

Data was analyzed using Epi info version 3.5.1. The software was used to generate frequencies, graphs, calculate relative risks (RR), attributable risks (AR), adjusted odds ratios (AOR) and their 95 % confidence intervals. Univariate and bivariate analysis was performed to check for associations between exposure and outcome variables. P-values less than 0.05 were considered statistically significant. Forward step-wise logistic regression analysis was performed to control for confounding and subsequently identify independent risk factors for contracting foodborne illness among workers.

Permission to conduct the study was sought and obtained local health authorities, the factory management and the health studies office (HSO) which is responsible for coordination of research activities in Zimbabwe. Ethical review was obtained from the Medical Research Council of Zimbabwe. Informed and written consent was sought from study participants. Confidentiality was maintained throughout and after the study. No names were included on completed questionnaires.

## Results

### Demographic characteristics of workers

A total of 98 workers were interviewed. Of these (87/98, 89 %) were males and (11/98, 11 %) were females. The majority of workers (77/98, 79 %) stayed in the urban area about 60 km away from the factory site whereas (21/98, 21 %) stayed in rural areas. Most workers had attained secondary education (49/98, 50 %) and tertiary education (46/98, 47 %). The A1-A3 grade had the majority (40/98, 41 %) of the study participants.

### Epidemic curve

The Epi curve showed that the index case was reported on September 14, 2012. The first symptoms occurred at around 2300 h, almost 11 h after food was served for lunch. Workers ate food from the factory canteen on September 14, 2012. Lunch was served between 12:30 PM and 14:00 PM and supper between 17:30 and 18:30 PM. Most cases occurred on September 16, 2012 between 12:30 and 0030 h (Fig. l). The mean incubation period was 48 h. The last day of onset was reported on the September 21, 2012.

**Fig. 1:**
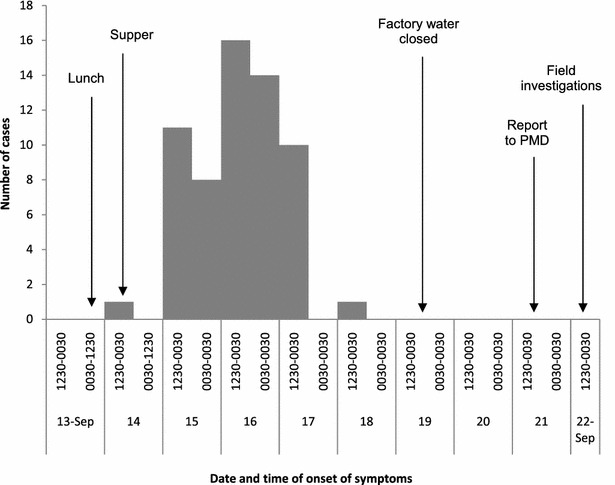
Date and time of onset of illness among cases (n = 98), food borne illness among factory workers, September 2012

### Risk factors for contacting foodborne illness

The overall attack rate was (58/98, 59 %) and of this (80/98, 82 %) were males and (8/98, 18 %) were females. There was no statistical difference in the attack rate between males and females p = 0.52. Eating beef stew [RR = 4.23, 95 % CI 2.15–8.31], vegetables [RR = 1.50, 95 % CI 1.04–2.14], Sadza [RR = 2.36, 95 % CI 1.24–4.49], on September 14, 2012 was significantly associated with foodborne illness. However drinking factory tap water [RR = 1.26, 95 % CI 0.88–1.80] was not significantly associated with food borne illness. Eating rice [RR = 0.17, 95 % CI 0.03–1.10] and fruit salads, [RR = 0.33, 95 % CI 0.06–1.90] respectively were less likely to be associated with foodborne illness (Table 1).

**Table 1:** Foods associated with illness among factory workers, September 2012

Food	Exposed			Not exposed			Relative risk	95 % CI
	Ill (58)	Not ill (40)	Attack rate (%)	Ill (58)	Not ill (40)	Attack rate (%)		
Beef stew	51	11	82	7	29	23	4.61	2.15–9.09
Sadza	51	23	69	7	17	29	2.36	1.24–4.49
Vegetables	37	16	70	21	24	47	1.50	1.04–2.14
Tape water	38	21	64	20	19	51	1.26	0.88–1.80
Rice	1	7	13	56	31	64	0.71	0.03–1.10
Salads	1	4	20	56	34	62	0.33	0.06–1.90

Workers who did not eat beef stew on September 14, 2012 were less likely to be ill from foodborne illness [RR = 0.16, 95 % CI 0.04–0.59]. Those who ate beef stew for either lunch or supper had a 4.45 times increased risk of illness compared to those who did not. Eating beef stew for both lunch and supper increased the risk of illness ninefold. The excess occurrence of illness among all the 431 workers was 63 per 100 (Table 2).

**Table 2:** Measures of impact for those who ate beef among factory works, September 2012

Ate beef stew	Ill	Not ill	Total	Attack rate/100	Attributable risk (AR)/100	AR %	Population attributable risk (PAR)/100	PAR %
Yes	51	11	62	82	63	77	41	68
No	7	29	36					
Total	58	40	98					

In multivariate analysis, workers who ate beef stew were more likely to experience [adjusted RR = 9.28, 95 % CI 2.78–30.91] foodborne illness when compared to those who did not eat beef stew.

### Microbiological analysis

*Klebsiella* spp. was isolated from beef stew and vegetables as well as in stool specimen collected from four (4/4, 100 %) patients. No serological and or bacteriocin typing was done. Swabs from inside refrigerator and drying rack had no growth of microorganisms. Of the four water sources from different points of the factory premises, water from reservoir tank had growth of viable microorganisms while untreated borehole water and canteen tape water had no growth of microorganisms.

### Clinical presentation among workers

**Fig. 2:**
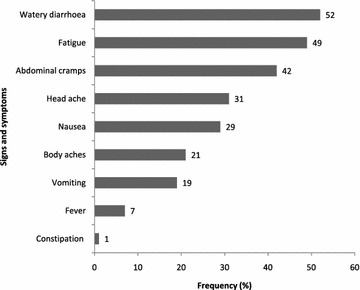
Clinical presentation of factory workers, Zimbabwe, September 2012

Diarrhoea was the most common presenting symptom (51/98, 52 %) followed by general body fatigue (48/98, 49 %). Abdominal cramps, headache, and nausea, constituted (41/98, 42 %), (30/98, 31 %) and (28/98, 29 %) respectively. Body aches, vomiting and fever were also notably present (20/98, 21 %), (18/98, 19 %) and (6/98, 7 %), (Fig. 2).

Majority (49/98, 50 %) of cases that reported to the factory clinic received metronidazole, oral rehydration solution (ORS) and paracetamol for the illness while some (17/98, 17.3 %) received zinc sulphate and doxycycline.

## Discussion

Contrary to our original hypothesis this study demonstrated that beef stew was associated with the foodborne illness at a factory in Gweru, Zimbabwe. All cases occurred within a single incubation period, confirming that the outbreak was caused by a common point source. Also the outbreak was self limiting suggestive of the presence of an enterotoxin producing microorganism. The outbreak highlighted that *Klebsiella* spp. has the potential to cause moderate to severe illness among health individuals.

In the present study, our main concern was to determine the risk factors for contracting the foodborne illness. For this purpose suspected food items, canteen surface swabs, swabs of food handlers and water samples were collected and examined. The data collected through questionnaires and microbiological analyses led to the conclusion that *Klebsiella* spp., detected in beef stew, vegetables and stool specimen of patients was the main aetiological agent of the outbreak. Nevertheless, other possible food contaminants including *E. coli* with similar symptoms and mean duration of illness could not be ruled out.

One possible explanation for the food contamination is that a food handler who prepared the food might have been harbouring the pathogens. Transmission of pathogens from food workers to the food they handle is implicated as a contributing factor in approximately 20 % of food borne illness outbreaks [7, 12]. Although workers have been implicated in outbreaks, there were always not aware of their infections, either because they were in asymptomatic phase before symptoms began or because they were asymptomatic carriers [13, 14]. It is therefore important to put mechanisms in place, where food handlers should be encouraged to report any illnesses and thus refrain from food preparation to prevent other food borne infections.

A number of studies have reported the association of beef stew and vegetables with enterobacteriaceae *Klebsiella* spp. Enterobacteriaceae count could be taken as an indicator of possible enteric contamination in the absence of coli forms [15]. Enterobacteriaceae are useful indicators of hygiene and post processing contamination of processed foods [16].

On the other hand, enterobacteriaceae occur as normal flora of the intestinal tract. They are widely distributed in nature and can be emitted from industrial effluents and dust. At the factory, the canteen is less than 200 metres away from production unit where there is dust. This might also explain why *Klebsiella* spp. were found in Sadza, beef stew and vegetables. In our study, the contribution of industrial effluents and dust could not be eliminated.

An outbreak of foodborne illness attributed to *Klebsiella* spp. was described by Horvath et al. [17] where a total of 190 people became ill. The symptoms of foodborne illness appeared 10–15 h after a common meal was eaten [18]. Although recent outbreaks attributed to *Klebsiella* spp. are scarce, recent studies show the presence of and isolation of *Klebsiella* spp. in meat and meat products from retail shops and restaurants that sell ready to eat foods. These studies reveal the potential of *Klebsiella* spp. to cause foodborne illness [7, 12, 19, 20]

In developing countries, hygienic standards during and after food preparation are very low and poor hygienic quality favour enterobacteriaceae growth and contamination. Our results indicate the necessity of high quality hygienic conditions during food preparation and storage. Another very critical food safety factor is the temperature at which food is stored before serving. It is important to note food served for lunch was also served for dinner. This may indicate that reheating of food at the factory canteen cannot be ruled out.

Enterobacteriaceae (*Klebsiella* spp.) are an indicator of post processing contamination by food handlers which was the most likely scenario in our case. Humans are the main reservoir for the enterobacteriaceae [17].

Other enterobacteriaceae cannot be eliminated because some of the sample results were not available. Even though, the results concurs with a study in South Africa by Nyenge et al. 2012, where *E. coli* and *Salmonella* species which are the common food pathogens were also not isolated in the implicated beef stew where *Klebsiella* spp. were isolated [12]. Contrary to our findings, a study in Nigeria by Bukar et al. [6] isolated other enterobacteriaceae such as *Staphylococcus* and *Proteus* in the same food where *Klebsiella* spp. was isolated [6].

To mitigate foodborne illness outbreaks will require the participation of different stakeholders including those of disease surveillance, consumer education, food handling and processing. This begins with measures being implemented to correct faulty food handling and preparation practices.

In Zimbabwe, surveillance systems are in place but the incidence of foodborne illnesses are often unreported or undetected and the considerable minimal epidemiological evidence on the incident and pathogens of foodborne illness caused by contaminated food. This results in limited data to quantify the magnitude of foodborne illnesses in the country.

*Klebsiella* spp. is a less suspected pathogen in foodborne illnesses. On the other hand, since it was a production oriented plant, workers were not willing to participate in the study and some areas were restricted to such an extent that some grades were replaced by workers from the next grade.

## Conclusions

The outbreak was a foodborne illness most probably caused by an enterotoxin producing microorganism. The isolation of Enterobacteriaceae in foods that are fully cooked is a good indicator of post-processing contamination or inadequate cooking in the junior canteen. The district as well as factory clinic was not prepared for the outbreak and the implicated beef stew was withdrawn from the canteen. Training of food handlers in food safety is highly recommended.
